# Loss of international medical experiences: knowledge, attitudes and skills at risk

**DOI:** 10.1186/1472-6920-7-47

**Published:** 2007-11-28

**Authors:** Corita R Grudzen, Eric Legome

**Affiliations:** 1 Robert Wood Johnson Clinical Scholars Program, University of California, Los Angeles, Los Angeles, California, USA; 2 Department of Emergency Medicine, New York University/Bellevue Hospital Center, New York, New York, USA

## Abstract

**Background:**

Despite the great influence International Medical Experiences (IMs) can have on young physicians and their impact on patients and communities, they are not offered in all training programs and are at risk of being reduced in some due to stringent guidelines for funding of graduate medical education.

**Discussion:**

IMs provide unique experiences in clinical, epidemiologic, cultural, and political arenas. From an educational perspective, they broaden a physician's differential diagnostic skills and introduce clinical entities rarely seen in the U.S.

Time spent in developing countries emphasizes the importance of community health and increases cultural and linguistic competence. Experience working with the underserved during an IM has been shown to increase interest in volunteerism, humanitarian efforts, and work with underserved populations both in the US and abroad. IMs also afford physicians the opportunity to learn about the delivery of health care abroad and are associated with an increase in primary care specialty choice.

**Summary:**

It is time for the leaders in graduate medical education to prioritize international health opportunities. Leaders in academic medicine can press for changes in reimbursement patterns at the national level or special funds for international electives. Hospitals can set up separate accounts to help finance resident salaries and benefits while abroad. Individual departments must be flexible with resident schedules to allow elective time. Medical students and housestaff can organize and lobby larger organizations such as the American Medical Association (AMA), the American Association of Medical Colleges (AAMC), and specialty groups to make IMs universally accessible.

## Background

International medical experiences (IMs) can change a young physician's life. Dr. Paul Farmer, a recipient of a MacArthur award [1], was inspired as a medical student and resident working in Haiti. He went on to establish a hospital in rural Haiti and co-founded an international non-profit organization that has pioneered humanitarian work on several continents. Dr. Albert Schweitzer, the 1952 Nobel Peace Prize recipient, chose a medical career after missionary work in Africa. Having obtained his medical degree, he founded his hospital at Lambaréné in French Equatorial Africa. International medical work has undoubtedly inspired countless others to lives of service.

Despite the influence IMs can have on young physicians (see Table 1), they are not offered in all training programs and are at risk of being reduced at some due to stringent guidelines for funding of graduate medical education. Currently, academic medical centers in the US that train residents are reimbursed by the federal government under both a direct and indirect Medicare adjustment payment to the hospital [2]. The hospitals lose the benefit of the Medicare adjustment when the resident is outside a training site that pays their salary. Because foreign institutions do not pay residents' salaries during international electives, hospitals lose that funding. While this funding varies based on hospital area and percentage of Medicare patients, in New York it is several thousand dollars per resident per month [3]. In response, some medical centers are now restricting or disallowing outside electives. Academic faculty, staff, and departments often raised funds to assist residents with travel and housing. Some medical centers were previously willing to underwrite the cost of these electives, but are now under increasing financial pressure not to continue. Although a majority of pediatric residency programs were interested in IMs based on a survey done in 1999, only 25% of programs had an international child health elective in place [4]. In this paper, we aim to describe the demonstrated benefits of IMs as well as the challenges and barriers to their more widespread implementation.

**Table 1:** Potential benefits of IMs

Introduction to rare clinical diseases
Improvement in clinical skills
Exposure to other health care delivery systems
Primary care career choice
Public health or health policy career choice
Commitment to underserved populations or rural areas
Increased cultural competence

## Clinical Knowledge and Skills

IMs provide unique experiences in clinical, epidemiologic, cultural, and political arenas. From an educational perspective, they broaden a physician's differential diagnostic skills and introduce clinical entities rarely seen in the U.S. How many medical students have seen measles? How many residents have managed organophosphate overdose? Doctors learn about preventable tropical diseases and clinical entities rare in the developed world. Residents on an international child health elective based in Latin America saw illnesses they had never seen before (18% of cases) and diseases at more advanced stages than in the US (6%) [5]. Recognition of these diseases is crucial for treating new immigrants and travelers returning from abroad. In developing countries, physicians must rely on their history and physical exam rather than laboratory and radiologic tests as they have fewer resources. Working side by side with local doctors, residents learn to use only essential resources, which has been shown to strengthen exam skills [6]. Without every test at their fingertips, doctors in training learn to provide rational and effective care using just a history, physical exam, and limited testing.

## Commitment to Underserved Populations

Young doctors also gain perspective through international work. Growing up in a resource-rich society, an American's first contact with extreme poverty is often abroad. Though some medical students and residents are exposed to poor patients within the US, not all training programs include such patients and the level of poverty is less extreme than in developing countries where many still die of hunger and curable communicable diseases [7]. Medical students and residents who participate in IMs see inequities firsthand through the lens of the health care system. Following the traditional trajectory, students' idealism and desire to work with underserved populations has been shown to decline as they progress from preclinical training through residency [8]. IMs are one way to prevent this decline. Medical students who participate in international electives show an increased interest in volunteerism, humanitarian efforts, and work with underserved populations both in the US and abroad [9,10]. Students also displayed more compassion towards the underserved and a heightened awareness of the social determinants of health. With increasing numbers of uninsured and enduring health disparities [11], the US needs more dedicated physicians interested in ending health inequities. Experience working with the underserved during an IM can produce a commitment to this type of service.

## Cultural Competency

Exposure to new cultures and religions is also important in a global physician's development. IMs have been shown to increase cultural competence. In addition to increasing idealism and enthusiasm, medical students who participated in an IM reported increased perceptions and values conducive to serving multicultural populations [12]. A physician's ability to recognize and respect cultural differences may lead to improved communication and compliance by patients. In ethnically diverse, disadvantaged patients, experiences with and perceptions of the health care system affect adherence to treatment [13].

## Comparative Health Systems and Health Policy

IMs also afford physicians the opportunity to learn about the delivery of health care abroad. Work in countries with a national health service, for example, provides an important contrast to the way health care is delivered in the US. If our current residents are to help shape the future of medical delivery systems, they must become versed in health care realities other than our own. Time spent in developing countries also emphasizes the importance of community health in addition to increasing cultural and linguistic competence [14]. Residents had increased awareness of public health measures after international experience. Participation increased residents' perceived value of many community health interventions, such as breastfeeding and oral rehydration [15].

## Ambassadorial Roles

In addition to the importance these rotations hold to medicine in the US, young physicians are important and effective ambassadors for our country. Similar to volunteers serving in the Peace Corps, young physicians could help promote a better understanding of Americans on the part of those served as well as enhance the volunteer's understanding of another culture [16]. In addition to learning from doctors and patients in host countries, young physicians have an ability to offer lessons from the US healthcare training and medical literature that may be inaccessible to foreign physicians.

## Primary Care Career Choice

But do international medical experiences affect career choice and practice? Increasing evidence proves international electives can impact medical students' choice of residency specialty. IMs can be used as a recruiting tool, as residents in family practice listed the presence of an IM as the most important factor for choosing one site over another [17]. In addition, participants in a three-week international elective were more likely to match in primary care and pediatrics [18]. They also chose to practice in more culturally diverse and underserved areas, and the majority choose careers in primary care [10,19,20]. Residents with exposure to international medicine were more likely to work in academics or public service than to pursue private practice [18]. Residents in an international health track often switched from subspecialty to general medicine during residency [21]. Nonetheless, physicians interested in primary care and public health may simply be more interested in international health. Thus, the effect of these studies may be due to association rather than causation. More research in this area could help determine the true benefits and potential harms of IMs.

## Barriers and Challenges

IMs need to be well-supervised and structured with clear goals and objectives to be effective. Medical students and residents with little clinical experience can do more harm than good if performing above their abilities in developing countries. In addition, particular attention needs to be paid to safety abroad. In a study done of Australian medical students who participated in overseas electives, 64% experienced a health problem, the majority being self-limited diarrheal diseases [22]. Some also reported assault and sexual harassment. Other potential risks include exposure to bloodborne pathogens, for which not all US-based international voluntary medical organizations even have policies [23]. Guidelines and training for all these safety issues should be addressed before departure. Another potential barrier to wider implementation of IMs might be time-constraints. Time spent abroad would take away from other learning opportunities in residency. If residents participate in an IM then residency directors must be sure it meets the specific goals and objectives of their training.

Many important stakeholders have already recognized the importance of global health. In increasing numbers, medical students are traveling abroad for international health experiences [24,25]. The Institute of Medicine has recognized the need for health care workers to help fight HIV/AIDS and other diseases common in the developing world. As a result, they recommended establishment of a federally funded US Global Health Service in 2005 [26]. It is time for the leaders in graduate medical education to prioritize international health opportunities. There must be support from other stakeholders for this to occur. Leaders in academic medicine can lobby for changes in reimbursement patterns at the national level or special funds for international electives. Hospitals can set up separate accounts to help finance resident salaries and benefits while abroad. Individual departments must be flexible with resident schedules to allow elective time. Medical students and housestaff can organize and lobby larger organizations such as the American Medical Association (AMA), the American Association of Medical Colleges (AAMC), and specialty groups to make IMs universally accessible. These measures will be one step to ensure that we don't lose international experience and all the knowledge, skills, and idealism that come along with it.

## Discussion

### Resident perspective: Corita Grudzen, M.D., M.S.H.S

As a medical student at the University of California, San Francisco School of Medicine, I gained invaluable experiences while pursuing research and clinical experiences abroad. I spent six months working in HIV prevention in Sao Paulo, Brazil. As a volunteer I learned that, unlike our system in the US, antiretrovirals were provided at no cost to all Brazilians with HIV. It was experiences like this one that motivated me to pursue a career that combines clinical practice with health policy and health services research. As a student on the infectious disease ward of Hospital Clinic in Sao Paulo, Brazil, I met my first patients with scrofula, Chagas disease, and Leishmaniasis. While these diseases are still rare in the US, I feel well equipped to treat patients who are recent immigrants or who have recently returned from abroad. As a resident in emergency medicine, I was privileged to attend an international conference in India, treat flood victims in Guyana, and provide basic health care in El Salvador. In a small village outside San Salvador, I learned the value of the physical exam and used conjunctival pallor to diagnose anemia. At Georgetown Public Hospital in Guyana, I treated diseases I had only read about in textbooks and was forced to make difficult decisions about rationing of care. Whether treating a patient with cerebral malaria or deciding whom not to intubate for lack of ventilators, I realized daily the unique experiences I was afforded while working abroad. In addition to these unique clinical and cultural experiences, I was exposed to a broad range of health systems unlike our own that I believe provide a good background for a career in health policy. While I realize I have had minimal impact on the countries to which I've traveled, these IMs have made me a more linguistically, culturally, and humanistically-minded physician. In addition to now being able to communicate with patients in Spanish and Portuguese and broadening my own differential diagnosis, I am motivated to improve our own health care system with lessons learned from other countries.

### Residency Program Director perspective: Eric Legome, M.D

As a program director I am privileged to see first hand the effect international electives have on our residents' lives and careers. I see how they improve their clinical care along with their ability to function in our highly diverse emergency department. I have witnessed multiple residents' careers and lives change due to the ability to practice abroad. For example, residents who spent international medical electives abroad are now improving emergency medical systems in developing countries as well as helping foster the development of the specialty overseas. Just as important, however, is the clear change I see in residents who now can understand why cultural and ethnic influences may predispose someone to a certain disease entity or hinder them from actively participating in care that we feel is essential to their well being. These experiences lead to better patient understanding, more culturally appropriate interactions and a greater ability to think on a global scale when constructing a differential diagnosis.

Unfortunately, I have also have seen throughout graduate medical education an eroding of the commitment to allow residents to gain these experiences, as these rotations come under assault from hospitals who wish to retain their graduate medical education funding. Most of us in academic medicine agree that hospital funding is essential and understand the financial pressures hospitals are under. However, if we wish to maintain our preeminence in medicine, it cannot be just in new medicines and technologies. The future physician must be able to understand and anticipate the problems of an increasingly diverse population of patients. If we do not understand where they came from and the unique ecological, sociological, and cultural factors that affect them, we will be unable to fully practice a holistic approach to their illness.

It will take a consortium of program directors, medical educators and administrators from hospitals and the federal government to deliver all the education and financial requirements needed to grow these opportunities (see Table 2). Program directors and educators must define and study the benefits in detail. They must lay out the specific aims and requirements to make international electives safe, educational and long lasting in their effect. It will take better research and scholarship to define what makes them worthwhile and what benefit they bring to our own population. Hospital and government administers must discuss the ability to support and fund these rotations. For individual hospitals it may bring a better ability to compete for top residents and enhanced reputation. This alone may be worth the financial commitment. The AAMC or individual Residency Review Councils could set up a committee to evaluate how it may enhance medical education. The ability to deliver culturally competent care can be enhanced or met by international rotations when a patient population is not diverse. Lastly, the government, by allowing a small portion of the direct and indirect graduate medical education payments to be applicable for IMs would significantly enhance the ability to residents to participate.

**Table 2:** Strategies to increase IMs

Change in funding pattern of graduate medical education
Development of Residency Review Council IM committees
Commitment by leading medical student, resident, and physician organizations
Policy statement by ACGME
Individual hospital commitment
Development of a nationally-funded global health service corps

## Summary

Despite the great impact international medical electives can have on physicians and those they serve, they are limited in availability due to the nature of graduate medical education funding. Young physicians gain valuable clinical skills and are exposed to important cultural and practice differences while working abroad. Most importantly, some may go on to make a contribution to the health of developing nations and underserved populations, and thereby make an impact in decreasing health disparities. The solution must be creative and complex and will involve commitment, research and funding to allow them to reach their maximum potential.

## Competing interests

The author(s) declare that they have no competing interests.

## Authors' contributions

CG and EL have both made substantial contributions to conception and design, have been involved in drafting the manuscript and revising it critically for important intellectual content; and have given final approval of the version to be published.

## Pre-publication history

The pre-publication history for this paper can be accessed here:


